# Identification of key transcription factors in caerulein-induced pancreatitis through expression profiling data

**DOI:** 10.3892/mmr.2015.3773

**Published:** 2015-05-12

**Authors:** DACHUAN QI, BO WU, DANIAN TONG, YE PAN, WEI CHEN

**Affiliations:** 1 Department of Surgery, The First Affiliated Hospital of Soochow University, Suzhou, Jiangsu 215006, P.R. China; 2 Department of Surgery, Shanghai Jiaotong University Affiliated Sixth People’s Hospital, Shanghai 200233, P.R. China

**Keywords:** caerulein, transcription factor, pancreatitis, differentially expressed genes

## Abstract

The current study aimed to isolate key transcription factors (TFs) in caerulein-induced pancreatitis, and to identify the difference between wild type and Mist1 knockout (KO) mice, in order to elucidate the contribution of Mist1 to pancreatitis. The gene profile of GSE3644 was downloaded from the Gene Expression Omnibus database then analyzed using the t-test. The isolated differentially expressed genes (DEGs) were mapped into a transcriptional regulatory network derived from the Integrated Transcription Factor Platform database and in the network, the interaction pairs involving at least one DEG were screened. Fisher’s exact test was used to analyze the functional enrichment of the target genes. A total of 1,555 and 3,057 DEGs were identified in the wild type and Mist1KO mice treated with caerulein, respectively. DEGs screened in Mist1KO mice were predominantly enriched in apoptosis, mitogen-activated protein kinase signaling and other cancer-associated pathways. A total of 188 and 51 TFs associated with pathopoiesis were isolated in Mist1KO and wild type mice, respectively. Out of the top 10 TFs (ranked by P-value), 7 TFs, including S-phase kinase-associated protein 2 (Skp2); minichromosome maintenance complex component 3 (Mcm3); cell division cycle 6 (Cdc6); cyclin B1 (Ccnb1); mutS homolog 6 (Msh6); cyclin A2 (Ccna2); and cyclin B2 (Ccnb2), were expressed in the two types of mouse. These TFs were predominantly involved in phosphorylation, DNA replication, cell division and DNA mismatch repair. In addition, specific TFs, including minichromosome maintenance complex component 7 (Mcm7); lymphoid-specific helicase (Hells); and minichromosome maintenance complex component 6 (Mcm6), that function in the unwinding of DNA were identified to participate in Mist1KO pancreatitis. The DEGs, including Cdc6, Mcm6, Msh6 and Wdr1 are closely associated with the regulation of caerulein-induced pancreatitis. Furthermore, other identified TFs were also involved in this type of regulation.

## Introduction

Pancreatitis is an inflammatory disease associated with auto-digestion of the gland. It is widely accepted that the injury is initiated within pancreatic acinar cells subsequent to premature intracellular activation of the digestive enzymes (1), which results in hydrops, hyperemia, hemorrhage and necrosis in the pancreas (2). In addition, acute pancreatitis may be associated with liver dysfunction and increased capillary permeability.

Caerulein is a polypeptide present in the brain and gastrointestinal tract, which has been demonstrated experimentally to result in pancreatitis. Caerulein administered via an intraperitoneal injection has been reported to induce acute edematous pancreatitis in mice, leading to the release of actin in pancreatic acinar cells, disruption to the transport of digestive enzymes and increased cellular permeability (3). In addition, several genes involved in this process have been identified: Cystathionine-γ-lyase serves an important pro-inflammatory role via nuclear factor κB activation in caerulein-induced pancreatitis (4); S-propargyl-cysteine protects caerulein-induced acute pancreatitis via its involvement in the slow release of endogenous hydrogen sulfide (5); and neutral endopeptidase (NEP) is anti-inflammatory in caerulein-induced acute pancreatitis, with acute inhibition of NEP contributing to increased substance P levels (6).

It has previously been demonstrated that the knockout of certain TFs may also result in inflammation. For example, Mist1 (an exocrine-specific TFs) is important for the successful differentiation and functioning of pancreatic acinar cells (7). Another study demonstrated that Mist1 knockout (KO) mice exhibited an altered stress response and increased sensitivity to caerulein-induced pancreatitis (8). In Mist1KO mice, multiple stress-associated genes, including c-Fos and c-Jun (together forming the activator protein 1 transcription complex), early growth response 1, immediate early response 1, 2 and 5 and numerous heat shock proteins failed to accumulate in pancreatic tissue, suggesting a deficit in the ability to activate the stress response (8). However, the regulatory mechanism of these genes remains unclear.

In the current study, microarray data from caerulein-induced wild type and Mist1KO mice were used to isolate the differentially expressed genes (DEGs). Key regulatory TFs were then screened from regulatory networks that were constructed by DEGs, combined with the TFs in the database. Comparisons between the sample groups allowed the identification of important TFs, which may aid in the elucidation of the regulatory mechanisms in Mist1KO mice and contribute to knowledge of the pathogenic mechanisms underlying pancreatitis.

## Materials and methods

### Microarray data

The gene profile GSE3644 was downloaded from the Gene Expression Omnibus (http://www.ncbi.nlm.nih.gov/geo/). This expression dataset was created by Kowalik *et al* (8), who treated pancreases from wild type and Mist1KO mice with caerulein or saline as a control and processed them for RNA analysis. Targets from three biological replicates in each sample were generated and the expression profiles were determined using GeneChip Mouse Genome 430 Array (Affymetrix, Inc., Santa Clara, CA, USA). The 12 samples were obtained from three pancreases from each group (wild type saline, wild type caerulein, Mist1KO saline and Mist1KO caerulein) were used and biological replicates were analyzed.

### Identification of DEGs

The t-test was used to analyze the gene expression profile in wild type and Mist1KO mice using the following formula (9):

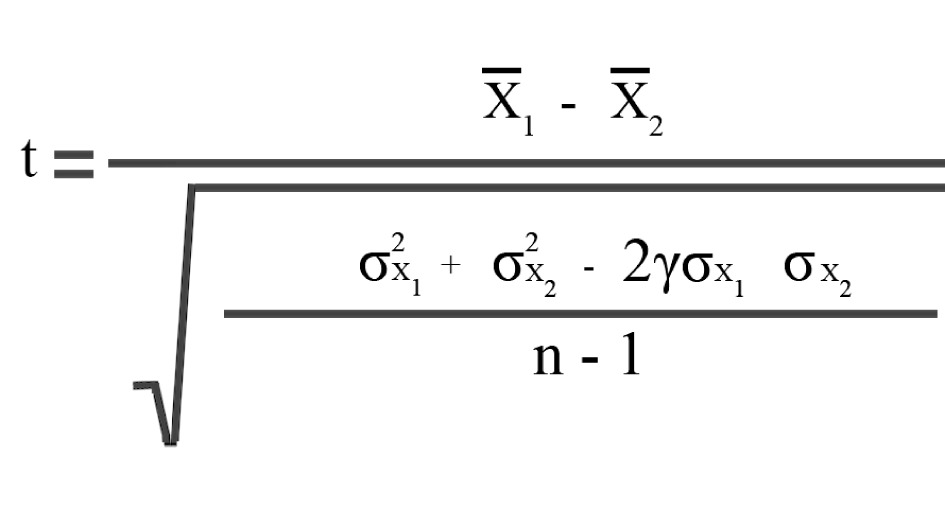


${\bar{X}}_{1}$ and ${\bar{X}}_{2}$ represent the average expression values of the gene in induced (caerulein-treated) and uninduced (saline-treated) groups; $\sigma_{X_{1}}^{2}$ and $\sigma_{X_{2}}^{2}$ are the variance of expression values under two different conditions; and γ represents the correlation coefficient for genes between the induced and uninduced groups. Genes with significantly different expression (P<0.05) were isolated and considered to be DEGs. The Bayesian method (10) was used to adjust the raw P-values into the false discovery rate (FDR).

### Construction of transcriptional regulatory network

A total of 34,679 transcriptional interaction pairs were isolated from the Integrated Transcription Factor Platform database (11), including 1,340 TFs and 4,785 target genes. Isolated DEGs were mapped into the transcriptional regulatory network, in which the interaction pairs involved at least one DEG were selected.

### Functional enrichment analysis

Gene ontology functional enrichment analysis was conducted, using Database for Annotation, Visualization and Integrated Discovery (DAVID; http://david.abcc.ncifcrf.gov/) online software (12). Furthermore, two-way hierarchical clustering was conducted for the identified DEGs and the samples to distinguish the function of these DEGs, applying the gplots (13) package in R language (http://cran.r-project.org/web/packages/gplots/).

### Enrichment analysis of gene sets

Fisher’s exact test was used to analyze the enrichment of target genes using the following formula (14):

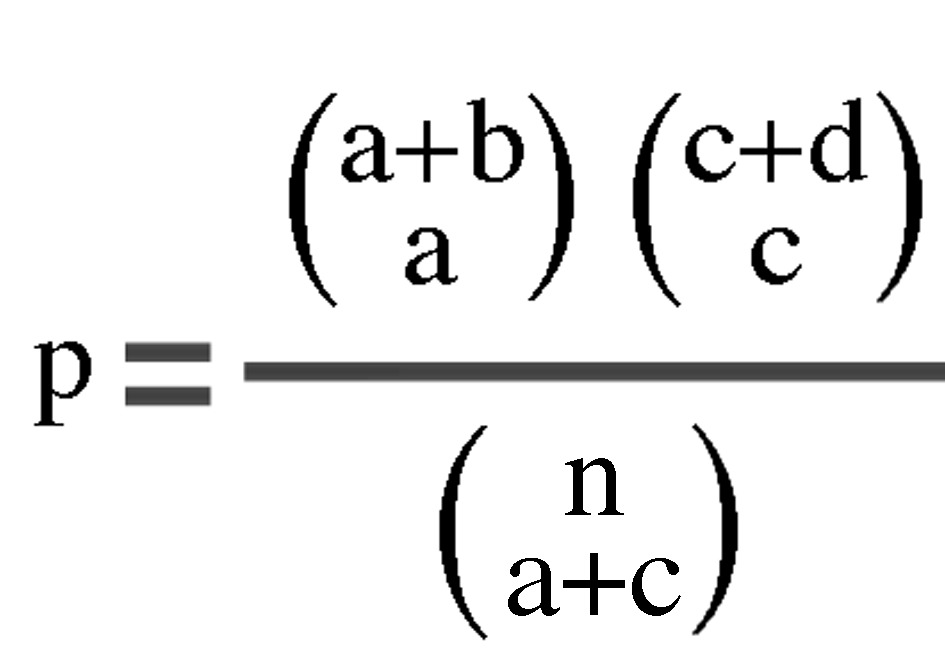


Where a represents a gene which is both a DEG and target gene of a TF; b represents a gene which is a target gene of a TF but not a DEG; c represents a gene which was a DEG but not a target of a TF; and d represents neither a DEG nor a target geen of a TF; n represents the sum of a, b, c and d.

## Results

### Identification of DEGs and enrichment analysis

**Figure 1:**
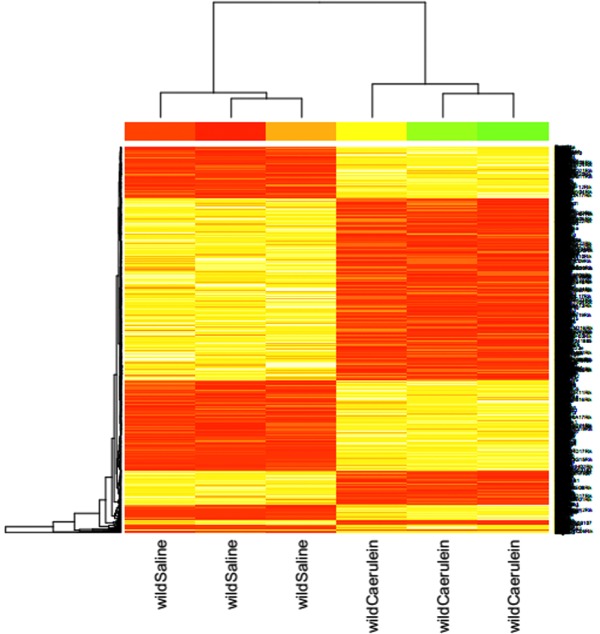
Two-way hierarchical clustering of differentially expressed genes in wild type mice. y axis, differentially expressed genes; x axis, samples.

**Figure 2:**
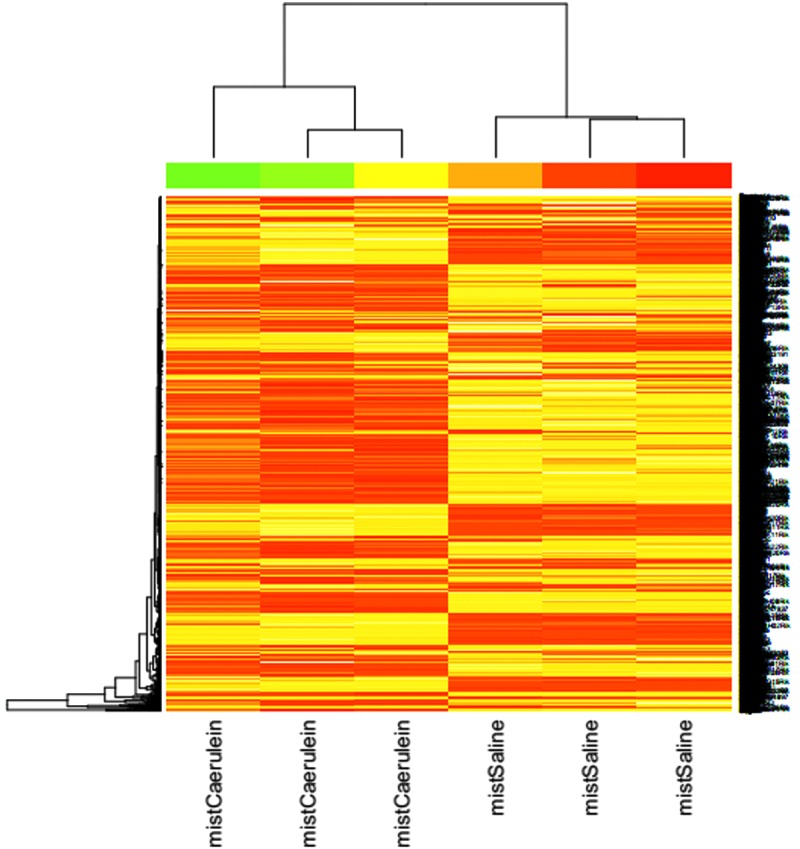
Two-way hierarchical clustering of differentially expressed genes in Mist1 knockout mice. y axis, differentially expressed genes; x axis, samples.

A total of 9,856 genes were detected and 1,555 DEGs were identified between caerulein-induced and non-induced wild type mice, whilst 3,057 DEGs between caerulein-induced and non-induced Mist1KO mice were identified, using the t-test. In order to verify an association between these DEGs and pancreatitis, functional enrichment analyses were conducted. As demonstrated in Tables I and II, DEGs in wild type mice were predominantly interrelated with the functions of the cell membrane, signal transduction in the vesicle and cell membrane and molecular transport, all of which have a clear association with dysfunction of the pancreas acini. In addition to the wild type enriched functions, DEGs identified in Mist1KO mice were involved in apoptosis, mitogen-activated protein kinase (MAPK) signaling pathways and cancer-associated functions. In order to verify the function of these DEGs, two-way hierarchical clustering was conducted to compare the DEGs between different treatment groups in the original samples. Consequently, distinct differences between the induced and non-induced groups in the wild type and Mist1KO mice were observed (Figs. 1 and 2).

### Construction and analysis of the transcriptional regulatory network

**Figure 3:**
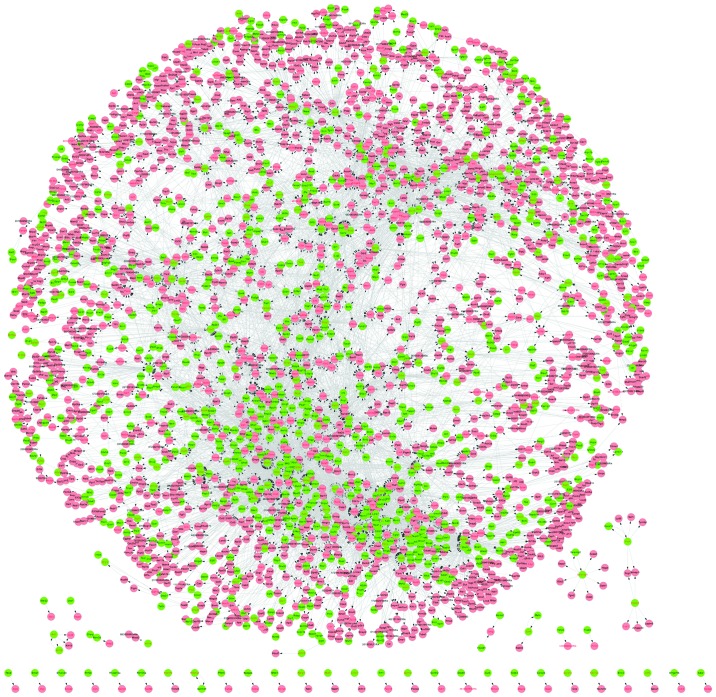
Transcriptional regulatory network of the differentially expressed genes in wild type mice. Green, transcription factors; pink, target genes.

**Figure 4:**
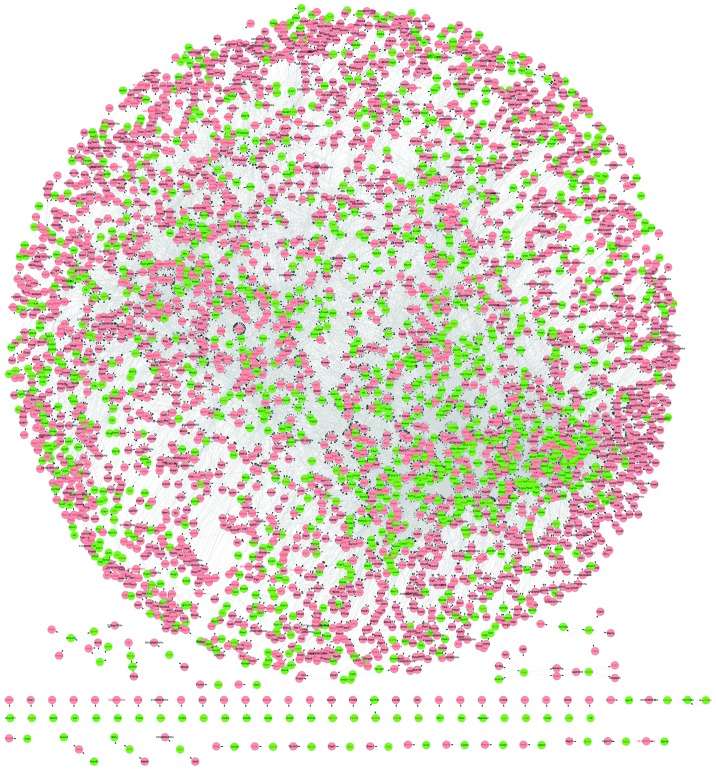
Transcriptional regulatory network in Mist1 knockout mice. Green, transcription factors; pink, target genes.

DEGs were mapped to the transcriptional regulation database, then a transcriptional regulatory network was constructed. As demonstrated in Figs. 3 and 4, there were 8,913 interaction pairs in wild type mice and 14,684 interaction pairs of the transcriptional regulatory network in Mist1KO mice. The network in Mist1KO mice was larger than that of the wild type, indicating that there will be complex regulatory events Mist1KO mice subsequent to injection of caerulein. In target gene sets regulated by TFs, the enrichment of DEGs was calculated. The greater the DEG enrichment in the target gene was, the greater the likelihood that the TF was associated with the pathogenicity of caerulein. According to the statistical analysis, a total of 952 TFs were identified in the regulatory networks. Subsequent to screening using a P-value criterion of 0.01, 188 TFs were identified to be closely associated with pathopoiesis in Mist1KO mice, whereas 51 TFs were identified in wild type mice. Functional enrichment with these TFs, indicated that the TFs in the Mist1KO mice were predominantly involved in the cell cycle and DNA replication and repair. In addition, TFs in wild type mice were involved in the cell cycle, cell adhesion and certain pathways associated with cancer. Among the top 10 TFs in each group, 7 TFs were observed in both types of mice (wild type and Mist1KO), which included S-phase kinase-associated protein 2 (Skp2); minichromosome maintenance complex component 3 (Mcm3); cell division cycle 6 (Cdc6); cyclin B1 (Ccnb1); mutS homolog 6 (Msh6); cyclin A2 (Ccna2); and cyclin B2 (Ccnb2). In the Mist1KO mice, minichromosome maintenance complex component 7 (Mcm7), lymphoid specific helicase (Hells) and minichromosome maintenance complex component 6 (Mcm6) were observed, whereas in wild type mice, WD repeat domain 1 (Wdr1), cyclin F (Ccnf) and X-box binding protein 1 (Xbp1) were observed (Tables III and IV).

## Discussion

Pancreatitis is a common pancreatic disease. Studies have demonstrated that caerulein may induce pancreatitis in mice and dysfunction in the Mist1 gene may enhance inflammation and increase the sensitivity to caerulein-induced pancreatitis (8,15,16). In the present study, a greater number of DEGs were identified between caerulein- and saline-treated Mist1KO mice compared with those observed in wild type mice. These DEGs were observed to be enriched in apoptosis, MAPK signaling and other cancer-associated pathways. Several key TFs were identified, a number of which were DEGs and may be able to regulate genes, such as Cdc6, Msh6, Wdr1 and Mcm6.

Cdc6 is an essential protein for the initiation of DNA replication (17), and may be highly prevalent in pancreatitis (18). It is reported that the induction of pancreatic acinar cell apoptosis is able to reduce the severity of pancreatitis (19,20). Thus, high expression levels of Cdc6 may lead to increased pancreatic acinar cell proliferation and pancreatitis. Msh6 forms a heterodimer with MSH2 to produce a mismatch recognition complex that functions as a bidirectional molecular switch to exchange ADP and ATP, as DNA mismatches are bound and dissociated (21). Although the involvement of Msh6 in pancreatitis has not been clearly defined, it is considered that Msh6 is involved in the regulation of pancreatitis via DNA mismatch repair (22). In the current study, Cdc6 and Msh6 were identified as DEGs in wild type and Mist1KO mice, further indicating the importance of these two genes in caerulein-induced pancreatitis.

Wdr1 encodes a protein containing nine WD repeats (involved in protein-protein interactions) (23) and was identified in wild type mice. The encoded protein may aid in inducing the disassembly of actin filaments (24), which is considered to affect the secretion of pancreatic acinar cells (25,26). Thus, Wdr1 is hypothesized to be involved in the regulation of pancreatitis through acinar cells.

In contrast, Mcm6 was observed in Mist1KO mice only. Mcm6, which belongs to the MCM family, is involved in the initiation of eukaryotic genome replication. The Mcm6 protein constructs an MCM complex with Mcm2, 4 and 7, which perform DNA helicase activity and may act to enzymatically unwind DNA (27,28). In addition, Mist1 is a basic helix-loop-helix TF and is involved in the maintenance of normal acinar cell function. A previous study reported that the expression of Mist1 results in a significant reduction in the proliferative potential of cells, whereas Mist1 knockdown leads to increased cell proliferation (7). Therefore, increased expression of Mcm6 may be due to the loss of Mist1 function, and thus enhances pancreatitis via increased proliferation.

In conclusion, high expression of Cdc6 and Mcm6 may lead to increased cell proliferation and pancreatitis. Msh6 and Wdr1 may be involved in the regulation of pancreatitis through DNA mismatch repair and acinar cells, respectively. Several key TFs that were not DEGs were also identified, these were considered to be involved in the regulation of caerulein-induced pancreatitis through target genes. Further investigation into these genes may contribute a more complete understanding of the regulatory network in pancreatitis and how this contributes to its pathogenic mechanisms.

## Figures and Tables

**Table I:** Top 20 significantly enriched GO terms of the differentially expressed genes in wild type mice (FDR<0.05).

Category	Term	FDR
GOTERM_CC_FAT	GO:0031090~organelle membrane	6.40×10^−08^
GOTERM_CC_FAT	GO:0005739~mitochondrion	1.59×10^−06^
GOTERM_CC_FAT	GO:0043233~organelle lumen	3.84×10^−05^
GOTERM_CC_FAT	GO:0031974~membrane-enclosed lumen	5.99×10^−05^
GOTERM_CC_FAT	GO:0070013~intracellular organelle lumen	9.99×10^−05^
GOTERM_CC_FAT	GO:0044429~mitochondrial part	1.09×10^−04^
GOTERM_CC_FAT	GO:0042470~melanosome	9.80×10^−04^
GOTERM_CC_FAT	GO:0048770~pigment granule	9.80×10^−04^
GOTERM_BP_FAT	GO:0022900~electron transport chain	0.001402
GOTERM_CC_FAT	GO:0031975~envelope	0.001806
GOTERM_MF_FAT	GO:0000166~nucleotide binding	0.001984
GOTERM_BP_FAT	GO:0007242~intracellular signaling cascade	0.002161
GOTERM_CC_FAT	GO:0031967~organelle envelope	0.003051
GOTERM_CC_FAT	GO:0031988~membrane-bounded vesicle	0.006726
GOTERM_CC_FAT	GO:0019866~organelle inner membrane	0.006783
GOTERM_CC_FAT	GO:0012505~endomembrane system	0.007954
GOTERM_CC_FAT	GO:0016023~cytoplasmic membrane-bounded vesicle	0.008693
GOTERM_CC_FAT	GO:0031982~vesicle	0.009787
GOTERM_CC_FAT	GO:0031410~cytoplasmic vesicle	0.016995
GOTERM_CC_FAT	GO:0005743~mitochondrial inner membrane	0.017543

FDR, false discovery rate; GO, gene ontology.

**Table II:** Top 20 significantly enriched GO terms of the differentially expressed genes in Mist1 knock out mice (FDR<0.05).

Category	Term	FDR
GOTERM_CC_FAT	GO:0005739~mitochondrion	9.65×10^−19^
GOTERM_CC_FAT	GO:0044429~mitochondrial part	6.55×10^−13^
GOTERM_CC_FAT	GO:0031090~organelle membrane	1.26×10^−11^
GOTERM_CC_FAT	GO:0031967~organelle envelope	2.94×10^−09^
GOTERM_CC_FAT	GO:0031975~envelope	3.85×10^−09^
GOTERM_CC_FAT	GO:0048770~pigment granule	3.06×10^−08^
GOTERM_CC_FAT	GO:0042470~melanosome	3.06×10^−08^
GOTERM_CC_FAT	GO:0031974~membrane-enclosed lumen	4.22×10^−08^
GOTERM_BP_FAT	GO:0008104~protein localization	6.11×10^−08^
GOTERM_CC_FAT	GO:0043233~organelle lumen	7.32×10^−08^
GOTERM_CC_FAT	GO:0005783~endoplasmic reticulum	7.53×10^−08^
GOTERM_CC_FAT	GO:0070013~intracellular organelle lumen	1.70×10^−07^
GOTERM_BP_FAT	GO:0015031~protein transport	3.98×10^−07^
GOTERM_CC_FAT	GO:0005829~cytosol	4.08×10^−07^
GOTERM_CC_FAT	GO:0031980~mitochondrial lumen	4.44×10^−07^
GOTERM_CC_FAT	GO:0005759~mitochondrial matrix	4.44×10^−07^
GOTERM_BP_FAT	GO:0045184~establishment of protein localization	6.95×10^−07^
GOTERM_MF_FAT	GO:0000166~nucleotide binding	6.38×10^−07^
GOTERM_CC_FAT	GO:0012505~endomembrane system	6.27×10^−07^
GOTERM_CC_FAT	GO:0019866~organelle inner membrane	4.02×10^−06^

FDR, false discovery rate; GO, gene ontology.

**Table III:** Top 10 disease-associated transcription factors (ranked by P-value) in Mist1 knockout mice.

Gene name	DEGs regulated (n)	Non-DEGs regulated (n)	Total DEGs of all transcription factors (n)	Total non-DEGs of all transcription factors (n)	P-value
Mcm7	54	265	3,750	5,788	1.69×10^−17^
Skp2	43	233	3,761	5,820	2.97×10^−17^
Mcm3	25	176	3,779	5,877	1.25×10^−16^
Cdc6	25	174	3,779	5,879	2.97×10^−16^
Ccnb1	20	154	3,784	5,899	1.14×10^−15^
Msh6	11	121	3,793	5,932	3.13×10^−15^
Hells	29	178	3,775	5,875	4.77×10^−15^
Mcm6	34	193	3,770	5,860	4.79×10^−15^
Ccna2	16	137	3,788	5,916	6.21×10^−15^
Ccnb2	15	132	3,789	5,921	1.38×10^−14^

P<0.05 was considered to indicate a statistically significant difference. DEGs, differentially expressed genes.

**Table IV:** Top 10 Disease-associated transcription factors (ranked by P-value) in wild type mice.

Gene name	DEGs regulated (n)	non-DEGs regulated (n)	total DEGs of all transcription factors (n)	total non-DEGs of all transcription factors (n)	P-value
Mcm3	11	190	1,547	8,109	8.60×10^−06^
Wdr1	46	107	1,512	8,192	8.92×10^−06^
Skp2	20	256	1,538	8,043	2.44×10^−05^
Ccna2	7	146	1,551	8,153	2.63×10^−05^
Cdc6	12	187	1,546	8,112	3.18×10^−05^
Ccnf	1	75	1,557	8,224	5.02×10^−05^
Ccnb2	7	140	1,551	8,159	5.15×10^−05^
Xbp1	15	18	1,543	8,281	5.47×10^−05^
Ccnb1	10	164	1,548	8,135	8.47×10^−05^
Msh6	6	126	1,552	8,173	9.38×10^−05^

P<0.05 was considered to indicate a statistically significant difference. DEGs, differentially expressed genes.
